# Isoorientin Inhibits Inflammation in Macrophages and Endotoxemia Mice by Regulating Glycogen Synthase Kinase 3*β*

**DOI:** 10.1155/2020/8704146

**Published:** 2020-10-27

**Authors:** Yingui Li, Yijing Zhao, Xiaoqin Tan, Jiayan Liu, Yingkun Zhi, Lang Yi, Shasha Bai, Qun Du, Qing X. Li, Yan Dong

**Affiliations:** ^1^Science and Technology Innovation Center, Guangzhou University of Chinese Medicine, Guangzhou, Guangdong Province, China; ^2^Department of Molecular Biosciences and Bioengineering, University of Hawaii at Manoa, Honolulu, Hawaii 96822, USA

## Abstract

Isoorientin has anti-inflammatory effects; however, the mechanism remains unclear. We previously found isoorientin is an inhibitor of glycogen synthase kinase 3*β* (GSK3*β*) *in vitro*. Overactivation of GSK3*β* is associated with inflammatory responses. GSK3*β* is inactivated by phosphorylation at Ser9 (i.e., p-GSK3*β*). Lithium chloride (LiCl) inhibits GSK3*β* and also increases p-GSK3*β* (Ser9). The present study investigated the anti-inflammatory effect and mechanism of isoorientin via GSK3*β* regulation in lipopolysaccharide- (LPS-) induced RAW264.7 murine macrophage-like cells and endotoxemia mice. LiCl was used as a control. While AKT phosphorylates GSK3*β*, MK-2206, a selective AKT inhibitor, was used to activate GSK3*β* via AKT inhibition (i.e., not phosphorylate GSK3*β* at Ser9). The proinflammatory cytokines TNF-*α*, IL-6, and IL-1*β* were detected by ELISA or quantitative real-time PCR, while COX-2 by Western blotting. The p-GSK3*β* and GSK3*β* downstream signal molecules, including NF-*κ*B, ERK, Nrf2, and HO-1, as well as the tight junction proteins ZO-1 and occludin were measured by Western blotting. The results showed that isoorientin decreased the production of TNF-*α*, IL-6, and IL-1*β* and increased the expression of p-GSK3*β in vitro* and *in vivo*, similar to LiCl. Coadministration of isoorientin and LiCl showed antagonistic effects. Isoorientin decreased the expression of COX-2, inhibited the activation of ERK and NF-*κ*B, and increased the activation of Nrf2/HO-1 in LPS-induced RAW264.7 cells. Isoorientin increased the expressions of occludin and ZO-1 in the brain of endotoxemia mice. In summary, isoorientin can inhibit GSK3*β* by increasing p-GSK3*β* and regulate the downstream signal molecules to inhibit inflammation and protect the integrity of the blood-brain barrier and the homeostasis in the brain.

## 1. Introduction

Isoorientin is a 6-C-glycosylflavone with a molecular formula of C_21_H_20_O_11_. It is present in many plant species, such as corn (*Zea mays*) silks and pollens, kudzu (*Pueraria tuberosa*), *Patrinia villosa*, [1–4]. Isoorientin exhibits antioxidant, antiviral, analgesic, antitumor, and anti-inflammatory activities [5–8]. Isoorientin reduces the development of inflammation in carrageenan-induced paw edema mice [6]. It enhances the activity of antioxidant enzymes, inhibits the release of inflammatory factors (IL-1*β*, IL-6, and TNF-*α*), and reduces liver oxidative damage and hepatitis in high-fructose-treated mice [9]. Isoorientin inhibits the activation of MAPKs and NF-*κ*B nuclear translocation in LPS-stimulated BV-2 microglia cells and consequently blocks the expression of inflammatory cytokines [10]. Isoorientin is a potential drug for the treatment of the inflammation-related diseases. The anti-inflammatory mechanism of isoorientin, however, has been unclear.

Our recent study showed that isoorientin is a substrate competitive inhibitor of GSK3*β* [11]. Overactivated GSK3*β* plays an important role in inflammatory response and the phosphorylation of the Tau protein, which is involved in the neurodegenerative pathological process [11]. Isoorientin reduces the hyperphosphorylation of the Tau protein and plays neuroprotective effects in SH-SY5Y cells [11]. Notably, GSK3 disorders are involved in a numbers of diseases, such as diabetes, reperfusion injury, mental stability, cancer, and neurodegenerative diseases [12–15], which are all related with inflammation. Sepsis leads to systemic inflammation and the destruction of homeostasis in the brain [16, 17]. Cognitive and memory impairments occur in rats or mice with endotoxemia in the open-field and Morris water maze experiments [18]. GSK3*β* plays an important role in inflammatory responses in endotoxemia mice [19]. Phosphorylation of GSK3*β* at the Ser9 site inactivates GSK3*β* [20–22]. GSK3*β* regulates its downstream signal molecules such as NF-*κ*B, ERK, Nrf2, and HO-1 in several or animal models [23–25].

The macrophage is the crucial part of the innate immunity system to trigger acute inflammatory responses. Although the anti-inflammatory properties of isoorientin have been revealed, the underlying mechanism remains indistinct. The present study was to explore the anti-inflammatory mechanism of isoorientin targeting GSK3*β* in comparison with LiCl that is a GSK3*β* inhibitor and also increases p-GSK3*β* [26, 27]. This study investigated the effects of isoorientin on the inactive form GSK3*β* (phosphorylation at Ser9) and its downstream signal molecules in macrophages, as well as the protective effect on the brain in endotoxemia mice.

## 2. Methods

### 2.1. Reagents

Isoorientin (HPLC purity ≥ 98%), LiCl, and LPS (from *Escherichia coli* 0111:B4) were purchased from Sigma (HPLC purity ≥ 98%, St. Louis, MO, USA). MK-2206 was purchased from Selleck Chemicals (Houston, Texas, USA). Monoclonal antibodies against p-GSK3*β*, GSK3*β*, p-ERK1/2, ERK1/2, COX-2, NF-*κ*Bp65, I*κ*B-*α*, HO-1, Nrf2, ZO-1, and occludin were purchased form Cell Signaling Technology (Danvers, MA, USA). The antibody against GAPDH was obtained from TransGen Biotech (Beijing, China). The horseradish peroxidase- (HRP-) conjugated anti-mouse and anti-rabbit IgG were purchased from MultiSciences (Hangzhou, China). Mouse TNF-*α*, IL-1*β*, and IL-6 ELISA detection kits were obtained from eBioscience (San Diego, CA, USA). ReverTra Ace qPCR RT Master Mix with gDNA Remover and SYBR® Green Realtime PCR Master Mix were purchased from Toyobo Co., Ltd. (Japan).

### 2.2. Cell Culture and Treatment

RAW264.7 murine macrophage-like cells purchased from the China Center for Type Culture Collection (Wuhan, China) were cultured in Dulbecco's Modified Eagle Medium (DMEM) (HyClonemao) containing 10% fetal bovine serum (FBS, Gibco) and antibiotics (100 U/mL penicillin and 100 *μ*g/mL streptomycin) in an atmosphere of 5% CO_2_ at 37 °C. Given the results of the growth curve, the cells were seeded at the density of 2 × 10^5^/mL. The cells were pretreated with isoorientin at different concentrations for 1 h, followed by stimulation with LPS (50 ng/mL) for an appropriate time.

### 2.3. Evaluation of the Effect of Coadministration of Isoorientin and LiCl

The anti-inflammatory doses were preliminarily defined. One hour after exposure to single or mixed LiCl and isoorientin, RAW264.7 cells were stimulated with LPS at 50 ng/mL for 8 h. The proinflammatory cytokine TNF-*α* in the supernatant was detected by ELISA. The interactions between isoorientin and LiCl were analyzed according to the published method of Jin's *Q* formula [28]. *Q* = *E*_*a*+*b*_/(*E*_*a*_ + *E*_*b*_ − *E*_*a*_ × *E*_*b*_), where *E*_*a*_, *E*_*b*_, and *E*_*a*+*b*_ represent the inhibition ratio of isoorientin, LiCl, and mixture of isoorientin and LiCl to TNF-*α*, respectively. *Q* < 0.85 suggests an antagonistic effect, 0.85 ≤ *Q* < 1.15 suggests an additive effect, and *Q* ≥ 1.15 suggests a synergistic effect.

### 2.4. Animals and Ethics Statement

The male BALB/c mice (6-8 weeks, 22 ± 2 g) were purchased from Beijing Huafukang Biotechnology Co., Ltd. (Beijing, China). The mice were adapted to the environment for 5 days prior to the experiment and were given food and drink randomly. The temperature of the room was 22 ± 2°C with a 12 h light/dark cycle. Animal care and treatment were performed in accordance with the Laboratory Animal Research Committee Guidelines of Guangzhou University of Chinese Medicine, Guangzhou, China.

### 2.5. Experimental Design and Animal Procedures

Mice were randomly divided into 5 groups (6 mice per group): control (saline), LPS (5 mg/kg), LPS (5 mg/kg) + isoorientin (25 mg/kg and 50 mg/kg), and LPS (5 mg/kg) + LiCl (100 mg/kg); isoorientin and LiCl were given by intragastric administration (ig) once a day for 5 days. Thirty minutes after the last administration of isoorientin or LiCl, LPS was injected intraperitoneally (ip) at a dose of 5 mg/kg. After 6 h of LPS injection, blood and tissues were collected. The blood was left at room temperature for 1 h and then centrifuged to obtain the sera.

### 2.6. ELISA for Cytokines

The levels of proinflammatory cytokines in sera or cell supernatants were measured by ELISA kits according to the instructions.

### 2.7. Western Blotting Analysis

The protein samples from RAW264.7 cells and mouse cortical tissue were lysed with RIPA lysis buffer (Kangwei Century Biotechnology, Beijing, China) containing protease and phosphatase inhibitors. Nuclear and cytoplasmic proteins of cells were obtained using a nuclear and cytoplasmic protein extraction kit according to the instructions (Kaiji Biotechnology, Jiangsu, China). Protein concentrations were measured by the BCA protein kit (TransGen Biotech, Beijing, China). Equal amounts of total proteins were separated by 10% sodium dodecyl sulfate-polyacrylamide gel electrophoresis and then transferred to PVDF membranes. The membranes were blocked with 5% nonfat dry milk in Tris-buffered saline containing 0.1% Tween 20 (TBST), washed with TBST, incubated with TBST containing primary antibodies(1 : 1000) and 5% bovine serum albumin (BSA) overnight at 4 °C, and subsequently incubated with TBST containing secondary antibodies (1 : 5000) and 5% nonfat dry milk at room temperature for 2 h. The enhanced chemiluminescence of protein blots was measured on a Multifunctional Imaging Analysis System (Bio-Rad, Hercules, CA, USA).

### 2.8. Quantitative RT-PCR (qPCR)

Total mRNA was isolated from tissues and was quantified. The purity and concentration of extracted total RNA were measured on a NanoPhotometer NP80 (Implen, Germany). The A260/A280 absorption ratio was between 1.8 and 2.2. Reverse transcription reactions were conducted according to the manufacturer's instruction of the ReverTra Ace qPCR RT Master Mix with gDNA Remover (Toyobo Co., Ltd.), followed by real-time PCR using the SYBR® Green Realtime PCR Master Mix (Toyobo Co., Ltd.). The primers and the product sizes were IL-1*β* (sense 5-TCCAGGATGAGGACATGAGCAC-3, antisense 5-GAACGTCACACACCAGCAGGTTA-3, product size 105 bp), TNF-*α* (sense 5-CAGGCGGTGCCTATGTCTCA-3, antisense 5-GGCTACAGGCTTGTCACTCGAA-3, product size 199 bp), IL-6 (sense 5-AGGATACCACTCCCAACAGACC-3, antisense 5-GCACAACTCTTTTCTCATTTCCAC-3, product size 101 bp), and GAPDH (sense 5-TGTGTCCGTCGTGGATCTGA-3, antisense 5-TTGCTGTTGAAGTCGCAGGAG-3, product size 150 bp). qPCR was performed on a 7500 Real-Time PCR System (Applied Biosystems, USA) as follows: 95°C for 30 s, 40 cycles of 95°C for 5 s, and 62°C for 30 s. The comparative Ct method (2^-*ΔΔ*Ct^) was used to analyze the relative expression of those genes by taking GAPDH as an endogenous control.

### 2.9. Statistical Analysis

SPSS 17.0 was used for statistical analysis. The data were expressed as mean ± SEM. The differences between experimental groups were analyzed with one-way ANOVA, while multiple comparisons were performed with the least significant difference (LSD) method. *P* < 0.05 was considered statistically significant.

## 3. Results

### 3.1. Isoorientin Inhibited Inflammatory Responses in LPS-Stimulated RAW264.7 Cells

**Figure 1:**
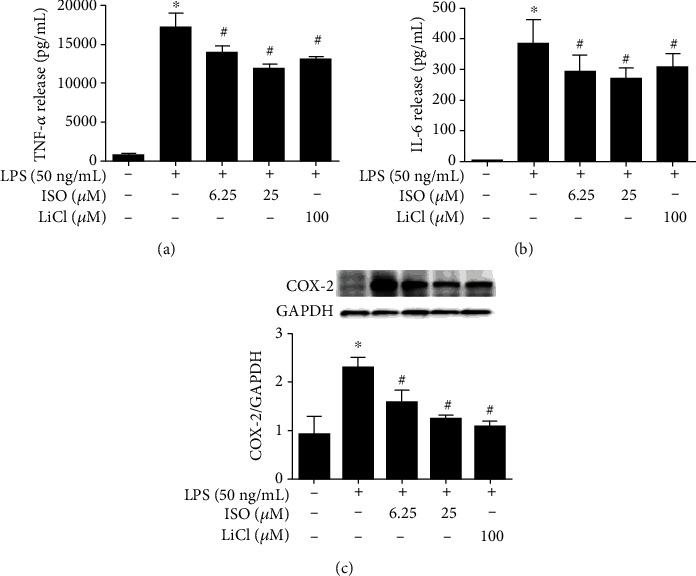
Effects of isoorientin on inflammatory cytokines and COX-2. RAW264.7 cells were treated with isoorientin (ISO) (6.25 and 25 *μ*M) or LiCl (100 *μ*M) for 30 min and then stimulated with LPS (50 ng/mL). After 8 h of stimulation, the cell supernatants were collected, and the levels of TNF-*α* (a) and IL-6 (b) were detected by ELISA (*n* = 4, 4 replicates). The cells were collected to measure the expression of COX-2 by Western blotting (*n* = 3, 3 independent experiments) (c). The data were expressed as mean ± SEM. ^∗^*P* < 0.05 versus the control group, #*P* < 0.05 versus the LPS group.

The secretion of inflammatory cytokines TNF-*α* and IL-6 and the expression of COX-2 were detected to illustrate the anti-inflammatory effect of isoorientin. Compared with the control group, LPS (50 ng/mL) significantly increased the secretion of TNF-*α* and IL-6 (Figures 1(a) and 1(b)) and the expression of COX-2 (Figure 1(c)), which were significantly decreased by isoorientin (6.25 and 25 *μ*M) and LiCl (100 *μ*M).

### 3.2. The Coadministration of Isoorientin and LiCl Showed an Antagonistic Effect

Our preliminary experiments defined that doses of isoorientin at 6.25 and 25 *μ*M and LiCl at 25 and 100 *μ*M exerted anti-inflammatory effects. Here, we investigated the effect of the coadministration of isoorientin and LiCl by analyzing the secretion of TNF-*α*. According to Jin's *Q* formula, *Q* < 0.85 showed an antagonistic effect, not an additive effect, in the coadministration group (Table 1). The results hinted that isoorientin might act on the same target with LiCl.

### 3.3. Isoorientin Increased the Expression of p-GSK3*β* in LPS-Stimulated RAW264.7 Cells

**Figure 2:**
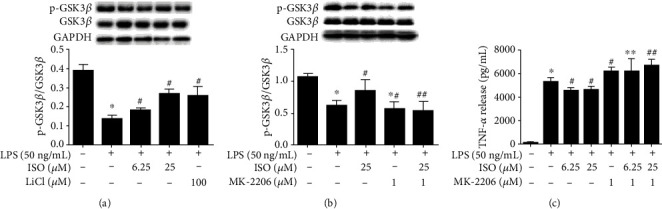
Effects of isoorientin on the phosphorylation of GSK3*β* (Ser9) and TNF-*α* in LPS-induced RAW264.7 cells. (a) Effects of isoorientin and LiCl on p-GSK3*β* and (b) effects of MK-2206 on p-GSK3*β*. RAW264.7 cells were treated with isoorientin (6.25 and 25 *μ*M) or LiCl (100 *μ*M) for 1 h without (a) or with (b) prior treatment of MK-2206 (1 *μ*M) for 30 min and then stimulated with LPS (50 ng/mL). After 30 min of LPS stimulation, the cells were collected for immunoblotting of p-GSK3*β*, GSK3*β*, and GAPDH (*n* = 3, 3 independent experiments). After 8 h of LPS stimulation, the cell supernatants were collected, and the levels of TNF-*α* were detected by ELISA (*n* = 4, 4 replicates) (c). The data were expressed as mean ± SEM. Statistical analysis was done using ANOVA. ^∗^*P* < 0.05 versus the control group, ^#^*P* < 0.05 versus the LPS group. ^∗∗^*P* < 0.05 versus the isoorientin 6.25 *μ*M group, ^##^*P* < 0.05 versus the isoorientin 25 *μ*M group.

The activity of GSK3*β* is inhibited upon its phosphorylation at Ser9 [20–22]. LiCl can increase the phosphorylation of Ser9. In Figure 2(a), LPS stimulation resulted in a significant decrease in the phosphorylation of GSK3*β* (Ser9) in RAW264.7 cells, whereas isoorientin (6.25 and 25 *μ*M) reversed the effects of LPS on p-GSK3*β*, similar to LiCl. The expressions of GSK3*β* were not affected significantly. The results suggested that isoorientin increased p-GSK3*β* to inhibit the activity of GSK3*β*.

### 3.4. MK-2206 Attenuated the Effects of Isoorientin on p-GSK3*β* and TNF-*α*

MK-2206 is a highly selective inhibitor of AKT. As previously reported, AKT is an important protein kinase to phosphorylate GSK3*β* [29]. In the present experiment, MK-2206 was used to inhibit the phosphorylation of GSK3*β* in RAW264.7 cells. MK-2206 pretreatment reversed the GSK3*β* phosphorylation induced by isoorientin (Figure 2(b)) but had no significant effects on the expressions of GSK3*β*. Isoorientin (6.25 and 25 *μ*M) decreased the production of TNF-*α* induced by LPS, while MK-2206 (1 *μ*M) attenuated the effect of isoorientin (Figure 2(c)). These results suggested that isoorientin inhibited the production of TNF-*α* by increasing the phosphorylation of GSK3*β*.

### 3.5. Isoorientin Regulated ERK, NF-*κ*B, and Nrf2/HO-1 Signaling Molecules

**Figure 3:**
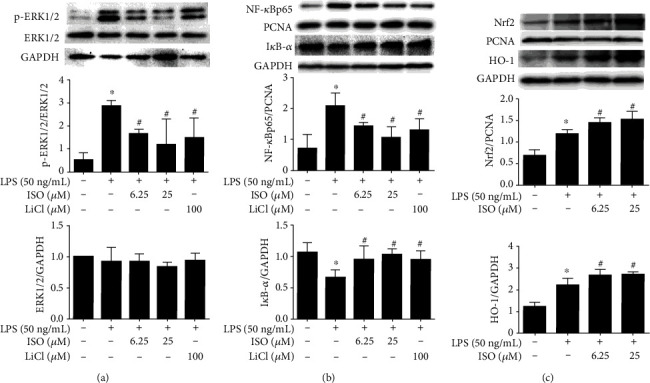
Effects of isoorientin on the activation of ERK (a), NF-*κ*B (b), and Nrf2/HO-1 (c) in LPS-activated RAW264.7 cells. RAW264.7 cells were treated with isoorientin (6.25 and 25 *μ*M) or LiCl (100 *μ*M) for 1 h with or without stimulation of LPS (0 and 50 ng/mL). After the indicated time of LPS stimulation, the cells were collected for immunoblotting of p-ERK1/2 and ERK1/2 (15 min), Nucl-NF-kBp65 (1 h), cyto-I*κ*B-*α* (1 h), Nucl-Nrf2 (18 h), and cyto-HO-1 (18 h). The data were expressed as mean ± SEM (*n* = 3, 3 independent experiments). Statistical analysis was done using ANOVA. ^∗^*P* < 0.05 versus the control group, ^#^*P* < 0.05 versus the LPS group.

It is well known that MAPK/ERK, NF-*κ*B, and Nrf2 participate in inflammatory responses, which are downstream signaling molecules of GSK3*β*. We wondered whether isoorientin regulated these downstream signaling molecules. Isoorientin (6.25 and 25 *μ*M) and LiCl (100 *μ*M) dramatically attenuated the phosphorylation of ERK1/2 (p-ERK1/2) in LPS-stimulated RAW264.7 cells, while the total protein of ERK1/2 remained unchanged in each group (Figure 3(a)). Isoorientin and LiCl also increased the expression of I*κ*B-*α* in the cytoplasmic fraction and suppressed the expression of NF-*κ*Bp65 in the nuclear fraction induced by LPS (Figure 3(b)), which suggested that isoorientin and LiCl inhibited the activation of the NF-*κ*B signaling pathway. The expression of Nrf2 in the nucleus and HO-1 in the cytoplasm increased upon LPS stimulation, while further increasing significantly upon isoorientin treatment (Figure 3(c)).

### 3.6. Isoorientin Inhibited Proinflammatory Cytokines in the Sera and Cortices of Endotoxemia Mice

**Figure 4:**
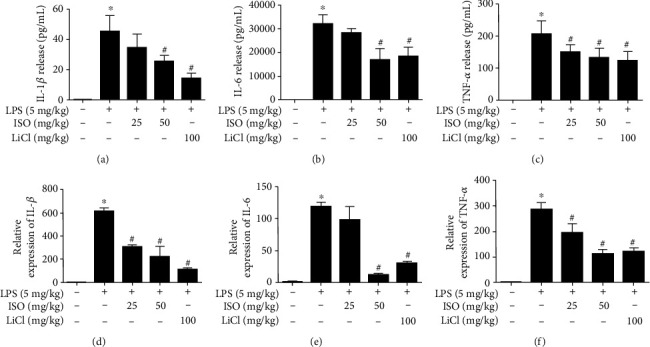
Isoorientin inhibited proinflammatory cytokines in the sera and cortices of endotoxemia mice. Isoorientin (25 and 50 mg/kg/d, ig) and LiCl (100 mg/kg/d, ig) were given once a day for 5 days, and after 30 min of the last administration, LPS (5 mg/kg, ip) was injected. The blood and tissues were collected after 6 h of LPS injection. IL-1*β* (a), IL-6 (b), and TNF-*α* (c) in the sera were detected by ELISA (*n* = 6, 6 biological replicates). IL-1*β* (d), IL-6 (e), and TNF-*α* (f) in the cortices were detected by qPCR (*n* = 3, 3 biological replicates). The data were expressed as mean ± SEM. Statistical analysis was done using ANOVA. ^∗^*P* < 0.05 versus the control group. ^#^*P* < 0.05 versus the LPS group.

The levels of IL-1*β*, IL-6, and TNF-*α* in the sera detected by ELISA and the mRNA levels in the cortices detected by qPCR increased dramatically in the endotoxemia mice. Isoorientin (50 mg/kg) and LiCl (100 mg/kg) inhibited significantly the production of IL-1*β*, IL-6, and TNF-*α* in the sera and in the cortices (Figure 4).

### 3.7. Isoorientin Increased Occludin and ZO-1 of Blood-Brain Barrier (BBB) Components in Endotoxemia Mice

**Figure 5:**
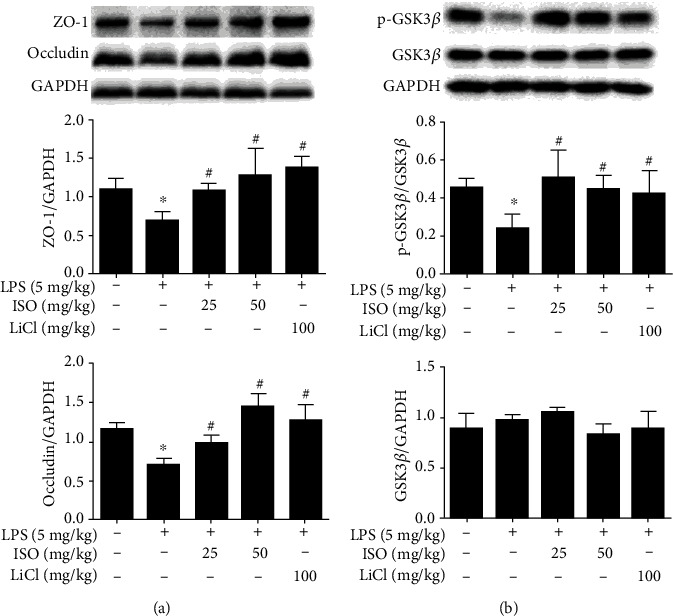
Effects of isoorientin on ZO-1 (a), occludin (a), and p-GSK3*β* (b) in the brain of endotoxemia mice. Isoorientin (25 and 50 mg/kg, ig) and LiCl (100 mg/kg, ig) were given once a day for 5 days, and after 30 min of the last administration, LPS (5 mg/kg, ip) was given. The brain tissues were collected after 6 h of LPS injection. Western blotting was used to measure the expression of ZO-1, occludin, p-GSK3*β*, and GSK3*β*. The data were expressed as mean ± SEM (*n* = 3, 3 biological replicates). Statistical analysis was done using ANOVA. ^∗^*P* < 0.05 versus the control group, ^#^*P* < 0.05 versus the LPS group.

Tight junction proteins, such as occludin and ZO-1, are the important components of BBB to perform normal functions. The occludin and ZO-1 decreased in the cortices of endotoxemia mice compared to the control, while isoorientin (25 and 50 mg/kg) and LiCl (100 mg/kg) increased the expressions of occludin and ZO-1 in endotoxemia mice (Figure 5(a)), which meant preventive effects of isoorientin on the disruption of BBB.

### 3.8. Isoorientin Increased the Expression of p-GSK3*β* in the Brain of Endotoxemia Mice

Isoorientin (25 and 50 mg/kg) and LiCl (100 mg/kg) increased the phosphorylation of GSK3*β* (Ser9) reduced by LPS (Figure 5(b)). The total protein of GSK3*β* did not change markedly among the groups. The results suggested that isoorientin increased p-GSK3*β* to inhibit the activity of GSK3*β* in the brain.

## 4. Discussion

GSK3*β* is a serine/threonine protein kinase. GSK3*β* plays an important role in regulating cellular inflammatory response, nerve, glucose metabolism, heart, and reproductive function [1, 12, 15]. Inhibition to the activity of GSK3*β* reduced prostaglandin E2, serotonin, histamine, and other inflammatory mediators in collagen II-induced rheumatoid arthritis in rats [30] and protected the nervous system from HIV-associated neurocognitive disorders [31]. GSK3*β* is a potential target for the treatment of immune diseases [32]. Phosphorylation of GSK3*β* at Ser9 (p-GSK3*β*) has a greater effect on GSK3*β* activity than phosphorylation at Tyr216 [20]. GSK3*β* is inactivated when phosphorylation occurs at Ser9 [20–22].

The PI3K/AKT signaling pathway plays an important role in regulating GSK3*β* activity via upregulating the phosphorylation of GSK3*β* (Ser9) in *Drosophila* and dorsoventral patterning in Xenopus embryos [33]. Modulating the PI3K/Akt/GSK3*β* signaling pathway affects the duration and intensity of the Toll-like receptor- (TLR-) mediated inflammation in septicemic shock [34]. GSK3*β* acts as an upstream molecule to regulate Nrf2 phosphorylation and Nrf2 detachment from the antioxidant response element (ARE). Parkinson's disease (PD) was alleviated by regulating the AKT/GSK3*β*/Nrf2 signaling pathway in a rat model of PD [23]. NF-*κ*B, a transcription factor with multiple transcriptional regulatory effects, is an important downstream pathway in the LPS-mediated inflammatory response signal transduction pathway in macrophages [35]. NF-*κ*B plays a key role in proinflammatory effects of GSK3*β* in human monocytes. The GSK3*β* inhibitor SB216763 inhibits the transcriptional activity of NF-*κ*B and reduces the production of inflammatory factors induced by TLR in human monocytes [25]. The MAPK signaling pathway regulates oxidative stress and injury response in cells. GSK3*β* knockdown blocks the IFN-*α*-induced phosphorylation of extracellular signal-regulated kinase (ERK) 1/2 (Thr202/Tyr204) in human Jurkat T cells [24].

Isoorientin is anti-inflammatory in carrageenan-induced paw edema mice and high-fructose-treated mice [6, 9]. It significantly blocks the inflammatory response in BV-2 microglia cells stimulated by LPS [10]. However, the anti-inflammatory mechanism of isoorientin has not been elucidated. In our previous study, isoorientin showed the ability of binding with GSK3*β in vitro* and reducing its activity in molecular docking and enzyme kinetics studies [11]. Lithium ion (Li^+^), an inhibitor of GSK3*β*, acts on GSK3*β* directly by competition with magnesium ion in the ATP binding pocket and has already been used for the treatment of bipolar disorders. Li^+^ also increases the phosphorylation at serine 9 of GSK3*β* to indirectly modulate GSK3*β* activity by activating Akt [26, 27, 36]. In the present study, we investigated whether isoorientin modulated inflammatory response by regulating p-GSK3*β* and exerted protective effects on brain injury.

In our study, isoorientin (≤100 *μ*M) and LiCl (100 *μ*M) had no inhibitory effect on the growth of RAW264.7 (data not shown). LPS (50 ng/kg) remarkably increased the release of TNF-*α*. Isoorientin at 6.25 *μ*M and 25 *μ*M decreased the expressions of proinflammatory cytokines and COX-2. In order to investigate the anti-inflammatory mechanism of isoorientin targeting GSK3*β*, TNF-*α* was detected to evaluate the effect of the coadministration of isoorientin and LiCl. The results suggested the coadministration exerted an antagonistic effect, but not an additive effect, indicating the same biochemical target of isoorientin and LiCl, namely, GSK3*β* (Table 1).

Furthermore, isoorientin upregulated p-GSK3*β*, similar to LiCl. Upregulation of p-GSK3*β* is well known to contribute to the inactivity of GSK3*β*. To verify the biochemical target of isoorientin, an AKT inhibitor, MK-2206 was used to inhibit the phosphorylation of GSK3*β*. Upon exposure to MK-2206 at 1 *μ*M (IC_50_ 12 *μ*M) alone, the viability of RAW264.7 cells did not alter dramatically (data not shown). MK-2206 (1 *μ*M) reversed the effects of isoorientin on TNF-*α* and p-GSK3*β* (Figure 2), suggesting inhibition of inflammation by isoorientin via upregulating p-GSK3*β*. The downstream signaling molecules of GSK3*β* were investigated in LPS-induced RAW264.7 cells. Isoorientin inhibited the activation of transcription factor NF-*κ*B and the ERK signaling pathway and activated the Nrf2/HO-1 signaling pathway (Figure 3).

Clinical studies have shown that sepsis patients have impairments in cognitive and memory functions [37, 38]. Endotoxemia mice show structural disorders in hippocampal neurons and cell necrosis in histopathological observations [39] and display symptoms such as disturbance of consciousness, abnormal behavior, and impaired sensory functions [40–42]. The BBB maintains immune privilege in the brain. Inflammation can increase the permeability of the BBB and disturb the homeostasis of the brain. IL-1*β*, IL-6, and TNF-*α* increased the permeability of BBB by downregulating tight junction proteins in endothelial cells [43]. Long-lasting inflammation damages the brain, which is common in neurodegenerative diseases [44, 45]. To clarify the protective effect of isoorientin on the brain, we detected the inflammatory cytokines in peripheral and central nervous systems, the BBB integrity, and the p-GSK3*β* (Ser9) in the brain of endotoxemia mice.

Anti-inflammatory doses of isoorientin and LiCl *in vivo* were determined according to previous studies [46, 47]. In the present study, LPS (5 mg/kg) significantly increased the levels of peripheral and central nervous system inflammatory cytokines, which were reduced by isoorientin (50 mg/kg) and LiCl (100 mg/kg) (Figure 4).

Occludin and ZO-1 are two key tight junction proteins in BBB, which determine the paracellular permeability to different ions or large molecules [48]. The decreased expression of occludin can be used as a marker of BBB damage [49]. Isoorientin (25 and 50 mg/kg) and LiCl (100 mg/kg) upregulated the expression of occludin, ZO-1 and p-GSK3*β* in the brain of endotoxemia mice (Figure 5), showing a good potential of reversing the destruction of BBB and treatment for GSK3*β*-related brain diseases. This study provided good evidence for clarifying the role of isoorientin in endotoxemia by protecting the BBB and regulating p-GSK3*β* in the brain.

Isoorientin binds with GSK3*β in vitro* and inhibits its activity [11]. Given that LiCl increases p-GSK3*β* to inactivate its activity, we found that isoorientin also increased p-GSK3*β*. These results suggested that isoorientin also regulated the upstream molecules of GSK3*β* to inhibit GSK3*β*. However, the merits of isoorientin superior to LiCl need further studies.

**Figure 6:**
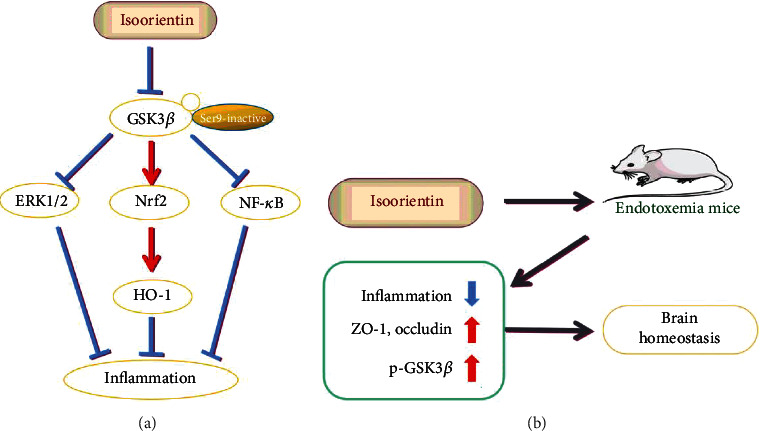
Proposed mechanism of isoorientin inhibiting inflammation in the monocyte macrophage RAW264.7 cells (a) and endotoxemia mice (b) via GSK3*β* regulation.

In conclusion, isoorientin increased the phosphorylation of GSK3*β* (Ser9) to inactivate its activity and regulated NF-*κ*B, ERK, and Nrf2/HO-1 signaling pathways to inhibit the production of proinflammatory cytokines and COX-2 in macrophages induced by LPS (Figure 6(a)). Isoorientin inhibited inflammatory responses in endotoxemia mice, increased p-GSK3*β* (Ser9), and protected the integrity of BBB by increasing the tight junction protein occludin and ZO-1 in the brain (Figure 6(b)). This was the first study to elucidate the anti-inflammatory mechanism of isoorientin from the perspective of GSK3*β* and to analyze the protective effect on inflammation-related brain injury.

## Figures and Tables

**Table 1:** Effects of coadministered isoorientin (ISO) and LiC1 in RAW264.7 cells (mean ± SEM, *n* = 3).

Compounds (*μ*M)	Inhibition (%) *E*_*a*_ or *E*_*b*_ ± SEM	Compounds (*μ*M)	Inhibition (%) *E*_*a*+*b*_ ± SEM	*Q* values
ISO 6.25	*E*_*a*_ 16.6 ± 6.5^a^	ISO 6.25+LiC1 25	10.1 ± 3.3	0.31
ISO 25	*E*_*a*_ 29.8 ± 3.6^a^	ISO 6.25+LiC1 100	27.6 ± 7.9	0.79
LiC 25	*E*_*b*_ 18.8 ± 3.3^a^	ISO 25+LiC1 25	28.8 ± 9.9	0.67
LiC1 100	*E*_*b*_ 21.7 ± 6.1^a^	ISO 25+LiC1 100	35.0 ± 9.3	0.78

RAW264.7 cells were treated with isoorientin (6.25 and 25 *μ*M) without or with LiCl (0, 25, and 100 *μ*M) for 30 min and then stimulated with LPS (50 ng/mL). After 8 h stimulation, cell supernatants were collected, and the levels of TNF-*α* were detected by ELISA. The data were expressed as mean ± SEM (*n* = 3, 3 replicates). ^a^*P* < 0.05 in comparison with the LPS group.

## Data Availability

The data used to support the findings of this study are included within the article, containing Table 1 and Figures 1–5. Other data that might be useful for the findings of this study will be supplied as supplementary information by the corresponding author (Yan Dong) upon request.

## Abbreviations

BBB: Blood-brain barrier
COX-2: Cyclooxygenase-2
ERK1/2: Extracellular regulated protein kinases
GAPDH: Glyceraldehyde-3-phosphate dehydrogenase
GSK3*β*: Glycogen synthase kinase 3*β*
HO-1: Heme oxygenase 1
ISO: Isoorientin
I*κ*B-*α*: Nuclear factor kappa B inhibitor alpha
LiCl: Lithium chloride
IL: Interleukin
LPS: Lipopolysaccharide
NF-*κ*B: Nuclear factor kappa B
Nrf2: Nuclear factor-E2-related factor 2
TNF: Tumor necrosis factor
ZO-1: Zonula occludens-1.
